# Neuropsychological and Psychiatric Features of Children and Adolescents Affected With Mitochondrial Diseases: A Systematic Review

**DOI:** 10.3389/fpsyt.2020.00747

**Published:** 2020-07-28

**Authors:** Elise Riquin, Philippe Duverger, Cindy Cariou, Magalie Barth, Clément Prouteau, Patrick Van Bogaert, Dominique Bonneau, Arnaud Roy

**Affiliations:** ^1^ Department of Child and Adolescent Psychiatry, University Hospital of Angers, Angers, France; ^2^ Mitovasc Unit, UMR CNRS 6015-INSERM 1083, Angers, France; ^3^ Laboratory of Psychology, LPPL EA4638, University of Angers, Angers, France; ^4^ Department of Biochemistry and Genetics, University Hospital of Angers, Angers, France; ^5^ Department of Pediatric Neurology, Angers University Hospital, Angers, France; ^6^ Reference Center for Learning Disabilities, Nantes University Hospital, Nantes, France

**Keywords:** mitochondrial diseases, children, adolescent, neuropsychological profile, psychiatric profile

## Abstract

Mitochondrial diseases (MDs) are a group of clinically heterogeneous genetic disorders that arise as the result of dysfunctional mitochondria. Only few medical articles deal with neuropsychological or psychiatric aspects of MDs.

**Aim:**

The present article aims to provide a systematic review of neuropsychological and psychiatric aspects of MDs.

**Methods:**

In order to identify all studies dealing with psychiatric and neuropsychological aspects of MDs in children and adolescents, we performed a search in the medical literature between April 2009 and April 2019 using PubMed, Cochrane, and Web of Science and we defined inclusion and exclusion criteria.

**Results:**

We found only seven studies that satisfy the inclusion requirements and criteria. The main psychiatric aspects reported in MDs were depressive and behavioral disorders. With regard to the neuropsychological aspects of MDs, developmental analyses showed an overall deterioration and developmental delay.

**Interpretation:**

Children and adolescents with MDs may present psychiatric symptoms and neuropsychological impairment. A more systematic investigation of psychiatric and neuropsychological features of MDs is needed to foster a better understanding of the phenotype of these diseases and their links with the genotype, which may have significant implications for the developmental trajectories of patients.

## Introduction

Mitochondrial diseases (MDs) are a group of clinically heterogeneous genetic disorders that arise as the result of dysfunctional mitochondria (1). The forms of MDs affecting children (<16 years old) have an estimated prevalence ranging from 5 to 15 cases per 100,000 individuals (1). MDs can be caused either by mutations in the mitochondrial DNA (mtDNA) or in genes from the nuclear genome encoding for mitochondrial proteins (2). Many MDs often involve multiple organ systems and typically affect organs that require the greatest amount of energy (i.e. brain, heart, muscles, kidney) (3–5). The main central nervous system (CNS) symptoms of MDs include epilepsy, hearing loss, visual impairment, intellectual disability, fluctuating encephalopathy, stroke-like episodes, ataxia, and spasticity.

Brain dysfunction in MDs can also result in neuropsychological or psychiatric disturbances including mood or behavior disorders, but few articles deal with this aspect of MDs (6, 7). Cognitive impairment is a common feature in adults presenting with mitochondrial encephalopathy, such as MELAS (mitochondrial encephalopathy lactic acidosis, and stroke-like episodes) (8) and psychiatric symptoms are associated with MDs in up to 70% of the adult population (9, 10). Along the same lines, a recent article suggested that mitochondrial activity could regulate the availability in neurons of GABA, an inhibitory neurotransmitter, leading to social deficits that can be rescued by the modulation of GABA level (11).

In contrast, only few articles have dealt with the neuropsychological and psychiatric features of children and adolescents with MDs (12–15). It is, however, crucial to consider this aspect of MDs in the pediatric population because the disease occurs in developing brains and may have not only a significant impact on developmental trajectories, but also may lead to psychological disturbances affecting quality of life.

## Methods

This review follows the 2019 Preferred Reporting Items for Systematic Reviews and Meta-Analyses (PRISMA) statements for performing a systematic review (16). As a first step, studies dealing with neuropsychological and psychiatric features of children and adolescents with MD were identified thanks to a computerized search in PubMed, Cochrane, and Web of Science using the following keywords: (mitochondrial disorder OR mitochondria OR mitochondrial cytopathy OR mitochondrial disease) AND (psychiatry OR psychiatric OR mental illness OR mental disorders OR major depression OR anxiety OR bipolar disorder OR schizophrenia OR psychosis OR neuropsychology OR cognitive OR executive function OR development OR IQ OR memory OR language OR learning abilities) AND (children OR adolescent OR pediatrics).

All articles found were registered using the bibliographic management software EndNote. Duplicates were checked not only digitally, but also manually in order to remove duplicates that could have been missed by the software.

In a second step, the articles previously found were selected using inclusion and exclusion criteria. Inclusion criteria included: 1) articles published in English; 2) articles reporting prospective and retrospective studies; 3) articles reporting longitudinal and cross-sectional studies; 4) articles reporting studies with an experimental group of children and/or adolescents with MD, and 5) articles published between April 2009 and April 2019.

Articles reporting results from adults, literature reviews, and case reports were excluded.

During the review phase of the articles, data was summarized by a primary reviewer (ER) who completed a data abstraction form. Then, a secondary reviewer (CC) checked the accuracy and completeness of the primary review. Finally, two other reviewers independently assessed the eligibility of the study for inclusion and unresolved disagreements between reviewers were adjudicated by a third reviewer (MB). The kappa coefficient, measuring the interrater reliability, was 0.84. Our local research ethics committee did not consider that its approval was needed for this systematic review.

**Figure 1:**
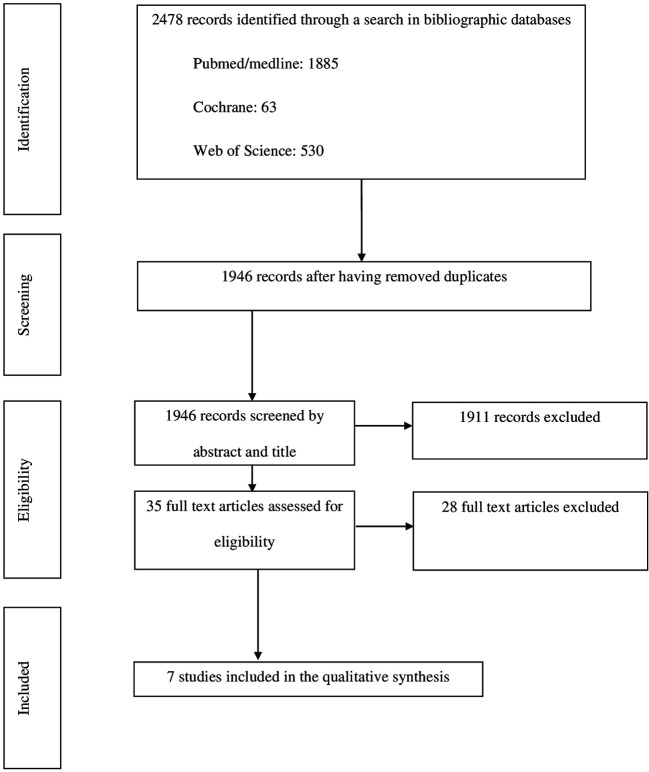
Preferred Reporting Items for Systematic Reviews and Meta-Analyses (PRISMA) flow diagram.

The PRISMA flow diagram of this bibliographic search is shown in **Figure 1**.

## Results

A total of 1,946 articles were retrieved during the first step of the review but only seven met the inclusion and exclusion criteria (**Table 1**). Two of these articles reported retrospective studies (21, 23), four reported prospective studies (17–20), and one reported a longitudinal study (22). The number of children included in these studies ranged from 14 (20) to 112 individuals (18). Two studies were carried out in the Netherlands (17, 19), two in the Republic of Korea (21, 22), two in the USA (20, 23), and one in the UK (18). Quantitative data obtained from psychometric tests were available in only six of these studies (**Table 1**) (19–23).

**Table 1:** Main results of the studies included in the literature review.

References, country of origin	Characteristics of the sample	Tests used	Results
Koene, (17), The Netherlands	35 children Inclusion criteria for MD: mutation in a mitochondrial (17) or in a nuclear (13) gene. 5 children with suspected depression were analyzed Age range: 2–18 years old, mean: 8.7 years old	Depression diagnosis (DSM-IV criteria) or HDRS for children older than 14 years old and/or Zung depression scale Holmes and Rahe Social Readjustment Rating Scale (Life Stress Event Scale)	Major depression (n=5; 14.3%) Psychotic symptoms (n=1; 2.9%) Life stress event score: increased susceptibility (>50%) in 3 patients.
Verity, (18), UK	2,493 children with PIND 112 (4, 5) children with MD Inclusion criteria for MD diagnosis: 112 suspected MD in the group of patients with PIND by a group of experts. Lactate levels increased in the blood and or CSF (n=87); muscle biopsy (n=75) and measures of mitochondrial respiratory chain enzymes: in 31 of these the result was diagnostic; DNA studies (n=50) yielded a specific diagnosis in 35; brain MRI (n=78) Age range: birth–14 years 7 months; median: 12 months 15 Leigh syndrome, 5 MELAS, 24 nonspecific, 68 other	No tests used Data were obtained from medical clinical observations	Developmental delay (n=43; 38.4%) Cognitive decline (n=1; 0.9%) Psychiatric symptoms: Autistic spectrum (n=3; 2.7%) Behavioral difficulties (n=1; 0.9%)
Morava, (19), The Netherlands	18 children who exhibit no developmental delays (individuals with IQ<70 were excluded) Inclusion criteria for MD: A clinical and biochemical diagnosis of OXPHOS disease for more than 1 year; muscle disease with developmental delay and variable systemic involvement. Control group: 18 children with inborn errors of metabolism 19 children with Sotos syndrome (a Mendelian disorder with non-progressive ID)	WISC (version not provided) CBCL -Anxious/depressed (1–18 years old), - Withdrawn/depressed (only in CBCL 6–18 years old) - Affective disorders (6–18 years old)/affective problems (1–5 years old). Comparison to American norms (sub-scale score of <65 is normal, 65–69 scores are borderline, and scores of >70 indicate a clinical problem)	Depressive behavior (n=7; 38.9%) CBCL, in the MD group: Compared to norms: Higher rate of withdrawn and depressive behavior (p = 0.0001) Higher levels of depressive behavior (p = 0.017) Compared to inborn errors of metabolism group: Anxious, depressive behavior in MD disorder: ns Withdrawn depressed behavior increased (p = 0.002) in the MD group Anxious depressive behavior: ns Compared to Sotos syndrome group: Withdrawn depressive behavior significantly higher in patients diagnosed with MD Anxious depressive behavior: ns
Schreiber, (20), USA	14 children were included Inclusion criteria for MD: selection *via* a website then parental questionnaire about how the MD was diagnosed (i.e. mutations identified or strongly suspected through muscle biopsy and/or blood test, in addition to clinical symptoms). Unspecified MD (n=5) Age range of children: not provided 7 male and 7 female adolescents/young adults. 5 of the subjects were between the ages of 19 and 21	BASC-2, for ages 12 to 21 years old BRIEF for older children/adolescents aged 13 to 18 years old or adult report for patients older than 18 years	Attitude to school associated with depression (p < 0.001), anxiety (P < 0.01), and internalizing problems (P < 0.01). Students mothers rated somatization more than a standard deviation above the mean (self-report: mean ¼ 62.21, SE ¼ 3.32; parent report: mean ¼ 78.86, SE ¼ 2.97). BRIEF results were not analyzed because of the age heterogeneity in the cohort
Eom, (21), South Korea	70 children Age at diagnosis: mean: 1.78 ± 2.52 years old Inclusion criteria for MD: biochemical enzyme assay of muscle tissues and modified mitochondrial diseases criteria (MDC) Inclusion criteria for nonspecific MD: no classical clinical symptoms, biochemical results, or mitochondrial DNA (mtDNA) deletions/duplications/point mutations that conform to known and established mitochondrial syndromes. 16 Leigh syndrome, 3 MELAS, 51 nonspecific	BSID-II WPPSI (Korean version) WISC-III (Korean version) Children’s adaptive Function: Social Maturity Scale (Korean version); Parent responses for 99-item CBCL (Korean version) for children 1.5–5 years of age or 118-item CBCL for children and adolescents 6–18 years of age Parental assessment (PSI; BDI for mothers)	Development (n=41): mental development = 52.9 ± 11.3 (=significant level of delay); psychomotor development = 52.1 ± 10.5 (=significant level of delay) IQ (n=15): FSIQ = 64.6 ± 21.8 (=mild levels of intellectual disability); VIQ=70.5 ± 19.2 (=mild levels of intellectual disability); PIQ = 58.6 ± 16.9 (=mild to moderate levels of impairment) Daily living function (n=54); social quotient = 50.2 ± 31.3 (significant impairments in adaptive function) Behavioral difficulties (n=28): CBCL = 62.9 ± 17.1; 43% above the cutoff; internalizing problems = 32%; externalizing problems = 25%; withdrawn = 46%; somatization = 11%; anxiety/depression = 29%; social problems 55%; cognitive problems = 20%; attention problems = 41%; delinquent behavior = 0%; aggressive behavior = 15%; emotional response = 31%; sleep problems = 38%; other problems = 20% PSI (n=32) = 88.6 ± 9.4; 75% above the cutoff BDI (n=26) = 14.6 ± 9.1; 65% above the cutoff
Eom, (22), South Korea	53 children Inclusion criteria for MD: biochemical enzyme assay of muscle tissue samples and meeting the modified MDC 11 Leigh syndrome, 2 MELAS, 40 nonspecific Age at diagnosis: mean: 3.12 ± 2.49 years old; lead time to diagnosis: 1.09 ± 1.15 years old (range, 0.6–1.46 years old)	KICDT DQ = (developmental age/chronological age) × 100] First visit prior to diagnosis, second visit prior to diagnosis, one-time diagnostic evaluation, and six post-diagnostic developmental evaluations	Pre-diagnosis evaluation (n=18): DQ = 60.4 ± 34.4 Diagnosis (n=37): DQ = 31.9 ± 25.8 Post-diagnosis (1 year after) (n=19): DQ = 27.8 ± 23.3
Shurtleff, (23), USA	49 children Age range: 61–250 months old Inclusion criteria for MD: biochemical testing, muscle biopsy with electron transport chain enzyme assay, and/or gene sequencing of mtDNA or nuclear genes involved in mitochondrial disease. When available, molecular genetic testing that confirmed pathological variants in either nuclear or mtDNA-encoded genes were obtained from Clinical Laboratory Improvement Amendments (CLIA) approved laboratories. All patients met clinical criteria of modified Walker criteria for diagnosing mitochondrial disease	WPPSI-III: 2½ to 7 years old WISC-III or -IV: 6 to 16 years old WAIS-III or -IV: 16 years of age and older FSIQ, VIQ, PIQ WMI or FFD PSI Vineland Adaptive Behavior Scales, 2^nd^ Edition	WISC-III or –IV (n=40) Vineland (n=9) = 85 [interquartile range (IQR): 50, 102] Group without seizures: FSIQ=100 (IQR: 86, 109), PIQ =100 (IQR 94, 112) Group with seizures: FSIQ = 67 (IQR: 49.5, 89), PIQ = 63 (IQR 54, 84) Statistical and clinical difference (Δ=33; 95% CI: 9, 52). Adaptive function measure = 43 (IQR: 37, 50) (patients with intractable epilepsy only)

Acronyms used: BASC, Self-report and parent report Behavior Assessment for Children; BDI, Beck Depression Inventory; BRIEF, Behavior Rating Inventory of Executive Function self-report and parent report; BSID, Bayley Scales of Infant Development; CBCL, Child Behavior Check List; CSF, cerebrospinal fluid; DQ, developmental quotient; FFD, Freedom From Distractibility Index; IQ, intelligence quotient; FSIQ, Full Score Intelligence Quotient; HDRS, Hamilton Depression Rating Scale; ID, intellectual disability; KICDT, Korean Infant and Child Development Test; MD, mitochondrial disorders; MDC, Mitochondrial Disease Criteria; MELAS, mitochodrial encephalopathy and lactate acidosis syndrome; OXPHOS, disorders of oxidative phosphorylation; PIQ, performance intelligence; PIND, progressive intellectual and neurologic deterioration; PSI, Processing Speed Index; VIQ, verbal intelligence; WAIS, Wechsler Adult Intelligence Scale; WISC, Wechsler Intelligence Scale for Children; WMI, Working Memory Index; WPPSI, Wechsler Preschool and Primary Scale of Intelligence.

### Psychiatric Features of Mitochondrial Diseases

Psychiatric disorders were analyzed in four articles, (17–19, 21) but only three of them (17, 19, 21) used rating scales to objectively assess them. The scales used in these studies were the Hamilton Depression Rating Scale (HDRS), the Zung depression scale (17), the Holmes and Rahe Social Readjustment Rating Scale (17), and the child behavior checklist (CBCL) (19, 21). These studies, the main results of which are shown in **Table 1**, primarily demonstrated the high prevalence of depressive symptoms and behavioral disorders, such as withdrawal, social problems, attention deficit, sleep disorders, and emotional response in children with MDs.

In the study performed by Eom et al. (21), 28 children with behavioral disorders were tested using the CBCL. The most severe features reported in this study were withdrawal, social problems, attention deficit, sleep disorders, and emotional response. In addition, a high level of parental stress was also reported. Similarly, almost 65% of the mothers from this study exhibited significant levels of depression.

Morava et al. (19) evaluated the psychological characteristics of 18 Dutch children with MDs who exhibited no developmental delays. In this study, a significantly high rate of withdrawal and depressive behavior was observed in the MD group in comparison to not only the normal population, but also to children affected with other forms of inborn errors of metabolism and to children with Sotos syndrome.

When comparing both these groups, the authors aimed to check whether factors such as the stress of being diagnosed with such conditions, fatigue, and discomfort were major determinants of the depressive behavior. This study showed a strikingly high incidence of depressive behavior in children with MDs, but the occurrence of depression was not significantly correlated with the degree of mitochondrial dysfunction and the clinical severity of the disease or, more specifically, with muscle involvement or severe CNS involvement. According to the authors, this lack of correlation supported the hypothesis that depressive behavior could be genetically determined, as shown in multiple studies (24), and that abnormal cerebral energy metabolism could be a risk factor for mood disorders.

In another study conducted in the Netherlands, Koene et al. (17) found that 5 children with MD out of 35 (14.3%) demonstrated major depressive behavior.

In a study conducted in the UK, Verity et al. (18) focused on the clinical presentation, the way the diagnosis was made, and the epidemiology of MD in children who exhibited progressive intellectual and neurological deterioration. The methodology was based on the British Pediatric Surveillance Unit (BPSU) card, which asks pediatricians to report children presenting progressive intellectual and neurological deterioration (PIND). The data reported by clinicians were classified and analyzed by an expert group. In this study, 3 children out of 112 had autistic spectrum disorder (2.7%) and 1 had behavioral difficulties (without specificities) (0.9%). In this study, however, no specific scales rating the psychiatric disorders were used.

### Neuropsychological Features of Mitochondrial Diseases

The neuropsychological profiles of children and adolescents with MDs has been analyzed in five studies (18, 20–23), the main results of which are shown in **Table 1**. However, only four of these studies used specific scales to evaluate the neuropsychological profiles of affected children.

Regarding intelligence, there was a substantial heterogeneity among juvenile patients with MDs with mild levels of intellectual disability and a Full Scale Intelligence Quotient (FSIQ) ranging from 64.6 to 100 in non-epileptic children (21, 23). By contrast, the cognitive profile appeared to be more affected in individuals with seizures and early onset of the disease (23).

In the study by Eom et al. (21), which included 70 children, several neuropsychological scales were used but not all measures were complete for all patients due to the limited functioning of some individuals. The results for 41 patients showed a significant level of mental and psychomotor developmental delay. Due to limited function, the intellectual quotient (IQ) measurement was performed for only 15 individuals and showed mild levels of intellectual disability and mild to moderate levels of impairment. Regarding overall development, children with MD showed an overall developmental delay and had consistently varying signs of cognitive decline.

Verity et al. (18) found that 43 children with MDs among 112 showed developmental delay (38.4%), one of them having a cognitive decline (0.9%). In this study, however, no specific scales were used and the data was only obtained from clinical observations.

In the study by Eom et al. (22), which included 53 children, the developmental function was evaluated at nine different time points (two points before diagnosis, one at the time of diagnosis, and six during the post-diagnosis phase) using the developmental quotient (DQ) from the Korean infant and child development test (KICDT), which assesses developmental age rather than chronological age. The overall DQ calculated for these 53 children showed a decline from the pre-diagnostic period to the post-diagnostic periods, suggesting an overall deterioration. However, even if declining patterns were consistently present, the characteristics of developmental deterioration were disparate. The authors described five phases: 1) pre-diagnostic initial decline phase; 2) pre-diagnostic accelerated decline phase; 3) post-diagnostic alleviated phase; 4) post-diagnostic reaccelerated decline phase; and 5) post-diagnostic stagnant phase. This study showed that diffuse brain atrophy, the clinical rating provided by the physician, and the age at which the first symptoms significantly affected the developmental level and decline. However, no significant effect was noted according to the type of syndrome and the severity of epilepsy, suggesting that these aspects may not directly reflect the developmental condition of the patients. The authors stressed some limitations in their study such as a preliminary study with a small number of patients, and the overall cohort that was not followed up consistently as they reviewed the data retrospectively.

Schreiber et al. (20) studied 14 children using the Behavior Rating Inventory of Executive Function^®^ (BRIEF^®^) scale to evaluate their behavior and executive functions. Unfortunately, the results were not significant because the age of participants in the cohort was too heterogeneous.

## Discussion

In this systematic review, we found only three articles dealing with the psychiatric features of children and adolescents affected by MDs and only five articles reporting their neuropsychological outcomes. These studies were conducted in only five countries (**Table 1**) and their methodologies were heterogeneous. For example, some studies took all the children in the sample into account (19–23) whereas others only analyzed the individuals who had psychiatric and behavior issues or had progressive intellectual and neurologic deterioration (17, 18). In two studies (17, 18), no specific psychometric scales were used and in another (20), data were not collected for all individuals in the sample.

### Psychiatric Features

The main psychiatric features affecting children and adolescents with MDs include depressive (14.3 to 38.9%) and behavioral disorders, such as withdrawal, social problems, attention deficit, sleep disorders, and emotional response (0.9 to 43%). This supports the fact that, even in children, mood disorders can be associated with abnormal cerebral energy metabolism (19). Koene et al. (17) stated that depression could affect 14.3% of their study group of patients with MDs in comparison to 3 to 4% of the adolescents from the general population. However, in this study, only on children with a history of depression were evaluated and not all the 35 children of their sample because many children were either too young or too severely affected. The authors therefore argued that the depression rate in children with MDs was probably underestimated (17). The prevalence of depression in MDs is similar to other chronic neurodegenerative disorders, although slightly higher, suggesting that the expression of major depression in these disorders is mostly dependent on the age of onset and clinical severity of the condition and regulated by the coping mechanism of the child and the family (25). Koene et al. (17) proposed the abnormal energy metabolism of the central nervous system as the underlying cause of the mood disorder in pediatric patients. Schreiber et al. (20) added that the etiology of depression and anxiety in young students with MDs is likely complex and may involve the biological and genetic substrates of these diseases. It is also well established that MDs lead to severe disabilities in most pediatric patients, significantly affecting their quality of life (26) and mood. In fact, having a chronic disease is a risk factor of depression (27–29). In the study by Verity et al. (18), the limitation is due to the fact that data were only obtained through clinical observations. The description of psychiatric symptoms is also very vague as “behavioral difficulties.” Moreover, this study was aimed to analyze children with progressive intellectual and neurological deterioration and not to take into account children with stable conditions. In addition, this study relied on information obtained from hospital records and on the diagnosis evaluation by pediatricians for children with developmental delay or autistic spectrum disorders. In the study by Eom et al. (21), the patient sample size was small and the data were collected retrospectively.

Anglin et al. (9) described cases of adults with MDs that was diagnosed many years after the onset of the psychiatric symptoms, which occurred during childhood or adolescence. These observations suggest that psychiatric symptoms could have been considered as early signs of the disease and could have led to an earlier diagnosis, especially if there were linked with other symptoms, such as a cognitive decline (30). The majority of cases in the literature had personal and family histories of multiple medical symptoms, including muscle weakness, hearing loss, fatigue, dysphagia, constipation, type 2 diabetes mellitus, migraines, and stroke-like episodes. From a psychiatric perspective, several patients had atypical aspects to their presentation and did not conform to strict DSM-IV diagnostic categories (9).

Beyond mood disorders, some authors described psychotic symptoms in adult populations (9) and Satogami (31) reported a case of Leigh syndrome who survived past adolescence and presented schizophrenia-like symptoms, including persecutory delusions and auditory hallucinations.

In addition, and in connection with analyses of adult populations, Rosebush (10) suggested that there could be a difference between sexes in the timing of onset of the psychiatric manifestations of inherited MDs, with girls potentially having earlier onset of the psychiatric manifestations.

The treatment of psychiatric illness in patients with MDs can be associated with resistance to treatment and even clinical deterioration. With some reported exceptions (32, 33), many psychotropic agents, including both typical and atypical anti-psychotic agents, selective serotonin reuptake inhibitors (SSRIs), and tricyclic antidepressants, have been found to impair mitochondrial function, generally through inhibition of complex I of the mitochondrial respiratory chain (10, 34). Nevertheless, it is challenging to delineate whether mitochondrial dysfunction occurs secondary to pharmaceutical treatment or whether it is a result of the underlying disease process itself (34). Therefore, intensive research is needed in psychiatric disorders to avoid malfunctioning of the mitochondria (35). In addition, as shown before, a very high proportion of patients is supposed to receive antiepileptic medications and could suffer from the side effects of antiepileptic treatment (decrease of vigilance, attention disorders, speech difficulties…). In this vulnerable population, additional iatrogenic symptoms should be carefully looked for and monitored over time.

### Neuropsychological Results

Regarding the neuropsychological consequences of MDs in the pediatric population, the evaluation of intellectual abilities is not consensual. Overall, the studies showed that many affected children have psychomotor and mental developmental delays (21) but the results of cognitive function evaluations are heterogeneous (20). The studies analyzed herein have many limitations. In the study by Eom et al. (22), the analyzes were retrospective and included an insufficient number of individuals to draw relevant conclusions. Moreover, this study presented only the pre-diagnostic neuropsychological profiles of the children together with comorbidities in their mothers. In addition, direct measurement of quality of life or long-term follow-up results were not provided.

The studies suggest deterioration of the DQ with a decline from the pre-diagnostic through the post-diagnostic periods (22). When such deterioration occurs, it may be either very quick and sudden, causing death or leading to a vegetative state, or slow and progressive (36). According to Shurtleff et al. (23), an early diagnosis of MD is essential to try to limit the cognitive loss with a more effective control of seizures. However, Shurtleff *et al*. (23) reported on only intelligence and adaptive functioning without distinction between the different types of MDs.

### Future Direction for Research

In the children and adolescents population, the difficulties, and in particular the psychiatric symptoms, must be identified and analyzed in a developmental logic. The disorders presented at a young age will have very different consequences from those presented in adult population, particularly in terms of learning abilities, but also quality of life. The issues of care and diagnosis are therefore particularly not the same as adult population. Future studies on neuropsychological and psychiatric features of MDs in children and adolescents should focus on the cognitive profiles and executive functions for which data are lacking. Executive functions are cognitive processes that assist the child in adapting their behavior to a specific purpose in a specific environment using reasoning, planning, and problem solving. Frontal-subcortical circuits are the effector mechanisms of executive functions and mature progressively throughout childhood and adolescence (37). Executive control function should be evaluated in children with MDs (20), considering that abnormal presence of intra-cerebral metabolites, such as lactate, could disrupt the development and the functioning of frontal-subcortical networks (20). Furthermore, the presence of these metabolites could also cause psychiatric symptoms, in particular in zones such as the caudate nucleus, the cingulate cortex, and the hippocampus with regard to depression and anxiety (38, 39). There is therefore a real interest in studying the association between neuropsychological aspects, executive functions, and psychiatric symptoms in children with MDs (40) The executive functions comprise all the processes that help to monitor and regulate cognitive processes during complex tasks and include planning, self-regulation, behavior organization, cognitive flexibility, working memory, error detection and correction, inhibition, sustained attention, and resistance to interference (41). Disorders of executive functions are also linked to anxiety and depression in children and adults (40, 42). Future studies should aim to understand the mechanisms underlying clinical symptoms in MDs in children and the adolescents by focusing on the analysis of the psychiatric profile and, more specifically, the anxiety and the depression and the neuropsychological profile through the dysexecutive syndrome hypothesis.

## Conclusion

This systematic review shows that children and adolescents affected with MDs may have psychiatric symptoms and neuropsychological impairment that are similar to those observed in adults highlighting the importance of considering them as key clinical signs for the diagnosis of MDs in pediatric practice. It seems of interest to better know the phenotype in children and adolescents and highlights the developmental specificities in MD diseases.

## Data Availability Statement

All datasets presented in this study are included in the article/supplementary material.

## Author Contributions

Design of the study: ER, CC, MB, AR. Data analysis: ER, CC, CP, AR. Article writing and revising: ER, PD, CC, MB, CP, PB, DB, AR. Revision of the manuscript: ER, PD, CC, MB, CP, PB, DB, AR.

## Conflict of Interest

The authors declare that the research was conducted in the absence of any commercial or financial relationships that could be construed as a potential conflict of interest.
